# Assessment of Freshwater Springs, Associated Diseases and Indigenous Perception in Ghizer, Gilgit-Baltistan, Pakistan

**DOI:** 10.12669/pjms.341.13956

**Published:** 2018

**Authors:** Shaukat Ali, Rubina, Sadiq Hussain

**Affiliations:** 1 Dr. Shaukat Ali, PhD. Department of Environmental Sciences, Karakoram International University Gilgit-Baltistan, Pakistan; 2 Ms. Rubina, MS. Department of Environmental Sciences, Karakoram International University Gilgit-Baltistan, Pakistan; 3 Dr. Sadiq Hussain, PhD. Department of Behavioral Sciences, Karakoram International University Gilgit-Baltistan, Pakistan

**Keywords:** Assessment, Spring water, Contamination, Associated disease

## Abstract

**Objective:**

To assess the quality of freshwater aquifers (springs) associated diseases, and indigenous perception in Ghizer, Gilgit-Baltistan.

**Methods:**

This was a cross-sectional study conducted at the department of environmental science, Karakoram International University Gilgit after approval from departmental research committee. In order to get the most accurate results, 18 water samples in triplicates were collected according to our lab own set of sampling standard operating procedures (SOPs) using pre-sterilized bottles of 100 ml from June 2016 to August 2016 along with associated diseases record and structured interviews from indigenous population. For physicochemical and microbial assessment, samples were tested before five hours after collection and associated diseases and indigenous perception was analyzed using descriptive statistical techniques.

**Results:**

Findings revealed that levels of pH, EC, phosphates, TDS, and nitrite, fall within WHO standards except nitrate, temperature and turbidity. The highest concentration (mgL^-1^± SE) of Hg in Barkolti and Barsat springs were (0.01±0.005) and (0.02±0.001) while Zn was (0.04±0.02) respectively. The concentration of Cu in Barkolti spring was (0.2± 0.03) while in Barsat spring below the detection level. The concentration of Cd, Pb, Cr, and Fe in both springs fall within WHO permissible limits. The mean value of *E. coli* recorded in both Barkolti and Barsat aquifers were (1.08cfu ±0.45) and (2.11cfu±0.56) respectively. The prevalence of water-associated diseases recorded in the order diarrhea, dysentery, typhoid, cholera, hepatitis and their incidence increases at high peak in summer. Irrespective of physicochemical and microbial components, indigenous population believed that the spring water has curative properties.

**Conclusions:**

Physiochemical and microbial parameters of spring's water fall within WHO standards except nitrate, turbidity, temperature, and *E. coli*, and incidence of certain associated diseases. However, indigenous population is unaware about the fact and believed that spring water very safe.

## INTRODUCTION

Quality of water is substantially more important than amount of water for keeping up a decent wellbeing and rural profitability.^1^ Environmental contaminants including physical, chemical and biological can enter the aquatic environment through naturally occurring events as well as anthropogenic activities, consequently can accumulate in the sediment and may be re-released into the overlying water through chemical, physical and biological processes.^2^

Freshwater aquifers (springs) are concentrated release of ground water showing up at the ground surface because of streaming of water. Springs are framed where the ground surface meets the water supply.^3^

Springs water are more contaminated and showed positivity of different bacteria like *E. coli, Total Coliform bacteria*, Salmonella, easily contaminate the exposed water through fecal, human and animal wastes on the land surface problems infiltration of contaminated surface water.^4^ Water may contain toxic inorganic chemicals, which may cause either acute or chronic health effects. Acute effects include nausea, lung irritation, skin rash, vomiting and dizziness, sometime death may occur. Chronic effects, like cancer, birth defects, organs damage, disorder of the nervous system and damage to the immune system are usually more common.^5^

Glacier and snow deposits are the principal sources of water in the northern areas of Pakistan for domestic purposes. People depend mainly on their irrigation channels for the supply of water for domestic consumption.^4^ Direct surface water and water supply schemes (based on surface water) are the common sources from where public fulfill their domestic water requirements. In district Ghizer, water channels, which bring water for irrigation, also serve for supply of water for domestic purpose. These channels are open and prone to contamination with feces and wastewater from residential area. In addition, clothes and domestic utensils are also washed in the same channels. To overcome uncertain and scare availability of water, pits are used for storage of water. The storage in pits facilitates removal of turbidity, but poor maintenance and cleaning of pits seems promoting bacterial contamination and is the major cause of unsafe water in the area.^4^ In Newfoundland, surveyed participants did not drink tap water because it is being perceived unsafe and around 23% of them prefer to use spring water despite the fact that it contain *E.coli* and coliforms 43% of the time.^6^

Therefore, the current study was conducted to assess the quality of water of selected springs water, associated diseases, and indigenous perception, as these aquifers are the main sources of the community for drinking and domestic purposes. The study findings would be useful to conduct awareness programs for community regarding quality of spring water and associated disease.

## METHODS

This is a cross-sectional study conducted from June 2016 to August 2016 after approval by departmental research committee, department of environmental sciences, KIU. A total of 18 samples in triplicates were collected from two springs of Ghizer; Barkolti and Barsat. Samples were randomly collected from upper, middle and lower altitudes where spring water connects with stream. Polythene pre-sterilized bottles of 100ml were used to collect samples. Samples were tested and analyzed before five hours after collection of samples for microbial study. The methods used for collecting samples and their analysis were in accordance with APHA (1992) guidelines.^7^

Data on water-associated diseases were collected from peoples' primary health initiative (PPHI) department, Ghizer from January to December 2015. To assess indigenous perception of spring water an open-ended question “*what do you think about spring water for your health*” was asked from 360 (180 women & 180 men) people belonging to the same area.

All the statistical computation was carried out using descriptive statistical techniques and Sigma plot (v.10) for the experimental and PPHI data and the results were expressed as (mean ± S.E). For indigenous perception data, first four answers to the question were recorded and analyzed by computing frequencies and percentages.

## RESULTS

In the present study; the mean values of nitrate, nitrite, pH, Zn, Fe, Cr, Cu, Cd and Pb were fall within national and WHO standards but temperature, turbidity, phosphate, Hg, and *E. coli* were above the WHO standards (Table-I).

**Table-I:** Physicochemical and microbial analysis (mgL^-1^± SE) of freshwater aquifers.

Parameters	Results		National Standards	WHO standard

	Barkolti	Barsat
Nitrate NO_3_(mg/l)	27.9 ±2.3	10.5±0.5	≤50	50
Nitrite NO_2_(mg/l)	0.2±0.05	0.3±0.06	≤3	3
Phosphate(mg/l)	3.3±1.9	10.6±0.3	0.02	0.02
pH	6.8±0.05	7.3±0.2	6.5 – 8.5	6.5 – 8.5
Temperature(°C)	17.7±0.6	18.6±0.6	-	<15 °C
Turbidity(NTU)	93.4±11.4	55.1±19.5	<5 NTU	<5 NTU
Zn(mg/l)	0.04±0.02	0.04±0.02	5.0	3.0
Fe(mg/l)	ND	ND	-	-
Cr(mg/l)	ND	ND	≤0.05	0.05
Hg(mg/l)	0.01±0.005	0.02±0.001	<0.001	0.001
Cu(mg/l)	0.2± 0.03	ND	2	2
Cd(mg/l)	ND	ND	0.01	0.003
Pb(mg/l)	ND	ND	≤0.05	0.01
E. Coli (cfu)	1.08 ±0.45	2.11±0.56	Exceed permissible limit (as detected in 100ml sample)	Exceed permissible limit (as detected in 100ml sample)

ND = Not Detected.

**Fig. 1:**
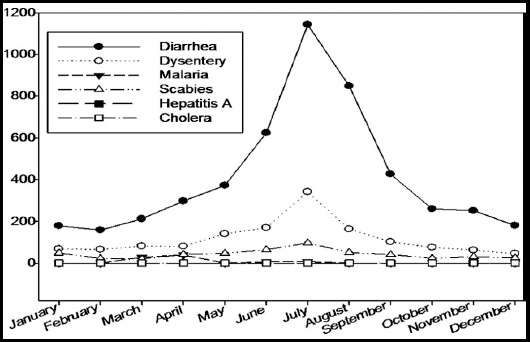
Monthly occurrence of water associated diseases.

PPHI one-year data revealed occurrence of water associated diseases. The most occurring disease was diarrhea (4949 (71.4%)) followed by dysentery (1391(20%)), scabies (510(7.4%)), and malaria (80(1.1%)), however, no cases of hepatitis and cholera were reported (Fig.1).

Results of indigenous perception of spring water is presented in Table-II, which revealed that all participants believed that spring water is good for health (has curative properties), most of them (90%) reported that it is good for digestive system, some of them (41%) reported that it is good for skin diseases, and very few (11%) claimed that it has healing power for all kind of diseases (Table-II).

**Table-II:** First four answers' frequencies regarding indigenous perception of spring water.

Answers to the research question	Frequency	%
1. Spring water is good for health	360	100%
2. Spring water is good for digestive system	324	90%
3. Spring water is good for skin diseases	148	41%
4. Spring water is good for all kind of diseases	40	11%

## DISCUSSION

*In the past there is no study available in the region that addresses the quality of drinking water, associated diseases, and indigenous perception. In the present study, some water quality parameters* fall within national and WHO standards while some parameters were exceeding the permissible limits. Similar findings were reported by other researchers from national as well as international context.^1^,^8^,^9^ Ince *et al*. claimed that when *E. Coli* exist in drinking water it may lead to water bound diseases like diarrhea, dysentery, scabies, malaria etc.^10^ same findings were shown by PPHI data where many cases of diarrhea, dysentery etc. have been reported in the study locale. Consistent findings were also observed in water complexes of District Gilgit where *E. coli* was detected due to waste of animals.^8^

The present study is the first attempt, which assessed people's perception of spring water in the selected areas of Gilgit-Baltistan but current debate in literature, evolves around people's preference for tap water or bottled water.^11^ Nowadays, in some areas, people prefer other sources of water such as artificially produced demineralized water and bottled water over tap water due to the identified link between water quality and human health.^12^ People preferred bottled water because of their dissatisfaction with tap water organoleptics, health related reasons, and other demographic and contextual factors.^11^ Others argue that, this is not always the case that bottled water have better quality than tap water.^13^,^14^ However, researchers reported that in the past people believed that good drinking water should be cold, nutritive, transparent, and portable irrespective of its biochemical quality.^15^ Similarly, in the present study we found that people perceived the spring drinking as having curative qualities due to being transparent irrespective of the presence of toxic elements and occurrence of associated diseases.

## CONCLUSIONS

*The present study was conducted to assess water quality, its associated diseases, and indigenous perception. Results shown the presence of some water contaminants and the occurrence of water bound diseases in the studied area but natives believed that the water is pure and has healing properties. Therefore, awareness programs are suggested to educate community about the presence of water contaminants and associated health risks*.

### Authors' Contribution

**SA** conceived, designed, did statistical analysis & editing of manuscript, takes responsibility for integrity of research work.

**R** did data collection, statistical analysis & manuscript writing.

**SH** did data collection, statistical analysis and editing of manuscript.
